# Supplementary material for: Rapid turnover of life-cycle-related genes in the brown algae
Source: Genome Biol. 2019 Feb 14;20:35. doi: 10.1186/s13059-019-1630-6 (PMC6374913; doi:10.1186/s13059-019-1630-6)
Supplement: Supplementary file 3 — Protein alignments, corresponding Gblocks html file and CDS sequences for the lineage conserved gametophyte-biased genes. (ZIP 2109 kb) [file 13059_2019_1630_MOESM3_ESM.zip › Con_GA_alignments/Ec-20_004590.aa_ali.fasta-gb.htm]

Ec-20\_004590.aa.fasta.trans.aa\_ali.fasta


## Gblocks 0.91b Results

Processed file: **Ec-20\_004590.aa.fasta.trans.aa\_ali.fasta**  
Number of sequences: **2**  
Alignment assumed to be: **Protein**  
New number of positions: **382** (selected positions are underlined in blue)

```
                         10        20        30        40        50        60
                 =========+=========+=========+=========+=========+=========+
Ec-20_004590     --MGVAS---EGQKDVVCP--DLIWTLNSGRSPGLDKLGPQQATNFFGTGSSCLTTKGRR
MSTRG.41506.1    MLRDVADGSDEAAAGVDCGSGKALFDNEGLPLSGLSLNKTTFENICLGHGNDSCDTRDEP
                                                                             


                         70        80        90       100       110       120
                 =========+=========+=========+=========+=========+=========+
Ec-20_004590     QVQTEPRAFPTKNGDHSYSTATSYG-----------------ELRMAR--LIPRPTSASP
MSTRG.41506.1    PADVGLRGLQGLRGDSPSCSRKGIDGLASRPSSAPARQCFCNELSDERPHRLSRNHATRA
                                                                             


                        130       140       150       160       170       180
                 =========+=========+=========+=========+=========+=========+
Ec-20_004590     LITPAQPMDGTWNMSPELTSRCTWRMAATRN--RAFTERFGLD--KPTCCTCPHGIPEGR
MSTRG.41506.1    REGPLHAGHGKFHSTAQLTAPGEWTLQKTKELWRGLVKIWGTSGFARSICQRTCGMRRGN
                    ##########################                    ########## 


                        190       200       210       220       230       240
                 =========+=========+=========+=========+=========+=========+
Ec-20_004590     GRTCGCVRQR-----EIELEAVQLCAALERRCPQWTMDGERNLWMIKPAGASRGQGVRVA
MSTRG.41506.1    HQNPDLQVQRFYNYVQLTTEAAQLCIALESRCPQWTMDGECNLWMLKPAGASKGRGVRVA
                                    #########################################


                        250       260       270       280       290       300
                 =========+=========+=========+=========+=========+=========+
Ec-20_004590     WTLDQILYAQKTMGGRVVQKYVETPLLLPRPSQQTRTP----------GDSRHTYLRSSR
MSTRG.41506.1    WTLEQILHAQETMGGRIVQKYVETPMLLSRCSPYPQLPEDSARGRGRRGDNCRRHLQLCR
                 ######################################          ############


                        310       320       330       340       350       360
                 =========+=========+=========+=========+=========+=========+
Ec-20_004590     DTPRA---GNVGSCAGELRLGEESDKGMSKADGVEAIGRGKIQILASLEAVDTQPLSSSG
MSTRG.41506.1    RATAPGERPGLGSCARRQGLGKEGDCRAPTVTEASAAQNKKEGCPPRLGVVTIAQTLPND
                            ##############          ################         


                        370       380       390       400       410       420
                 =========+=========+=========+=========+=========+=========+
Ec-20_004590     ETTSPPRSPEGQGDDTSYHSKHPTNNVDTNSGVFAEARKFDIRTWVLVTGWSPLEAFVFD
MSTRG.41506.1    EVVSLSRSP-------AYRMRR----MGIISRESSAAKKFDIRVWVLVTSWTPLEAFVFD
                                               ##############################


                        430       440       450       460       470       480
                 =========+=========+=========+=========+=========+=========+
Ec-20_004590     ECYLRVCPQSFTLAESRLGDQGVHLTNLSVRRPVKTYGRRRRQGHTRRPSSASTTGLRVV
MSTRG.41506.1    ECYVRVCPETFTLTESKFTDPGVHITNLSVRRPQP----RWRETKRLRP-STTTSACRAA
                 #################################      ########## ##########


                        490       500       510       520       530       540
                 =========+=========+=========+=========+=========+=========+
Ec-20_004590     SHGGGGALAHLSEDLPAQGPVGQRRDPRSRDEWDAG----EGVVGTQAELIQTLGGLIGQ
MSTRG.41506.1    IAKDGNTLGGRTTDVGNRKFPVDNKDARCHGEHNEGKEDTEGVVASQAELIRRLGEMKGG
                 ##############           ###########    ####################


                        550       560       570       580       590       600
                 =========+=========+=========+=========+=========+=========+
Ec-20_004590     RRVEKSTRSREEDVRTRGERLWRNKVSPSIDRVIGSTLLAAQPYVRPRASSFQLFGFDLH
MSTRG.41506.1    KRKGRSDGQEAEKKHISGERLWKNKVFPAIEGVVRSTLLAAQSFMKPRALSFQLFGFDLL
                 ############################################################


                        610       620       630       640       650       660
                 =========+=========+=========+=========+=========+=========+
Ec-20_004590     LDCDLNPWLLEVNLSPALSHTSKRQSAIIRSMCEGLLRLTVDRMFPALQPPSSCGGVDPE
MSTRG.41506.1    LDKDLNPWLLEVNQSPALTPSSKRQGALIASMCEGLLX----------------------
                 #####################################                       


                        670       680       690       700       710       720
                 =========+=========+=========+=========+=========+=========+
Ec-20_004590     SNDSDSLKQAEDQHCSGKWRPICPSTVGHESVVDVGQSIGRPSAEVLSLRGQHLTKKGLA
MSTRG.41506.1    ------------------------------------------------------------
                                                                             


                        730       740       750       760       770       780
                 =========+=========+=========+=========+=========+=========+
Ec-20_004590     RAERGVREATAANVLRAWWLSRADECRETRSKKSHAIAEISPWIIRLIARRRRRALQAKK
MSTRG.41506.1    ------------------------------------------------------------
                                                                             


                        790       800       810       820       830       840
                 =========+=========+=========+=========+=========+=========+
Ec-20_004590     ERSVRVIQAGWRRASARMLETRSHIARVAVCVVLQTMWRRRKLERCYRFRQTRCKVSSGL
MSTRG.41506.1    ------------------------------------------------------------
                                                                             


                        850
                 =========+====
Ec-20_004590     RRALQHAGYAARIH
MSTRG.41506.1    --------------
```

```
Parameters used
Minimum Number Of Sequences For A Conserved Position: 2
Minimum Number Of Sequences For A Flanking Position: 2
Maximum Number Of Contiguous Nonconserved Positions: 8
Minimum Length Of A Block: 10
Allowed Gap Positions: None
Use Similarity Matrices: Yes
```

```
Flank positions of the 11 selected block(s)
Flanks: [124  149]  [170  179]  [200  278]  [289  300]  [312  325]  [336  351]  [391  453]  [460  469]  [471  494]  [506  516]  [521  637]  

New number of positions in Ec-20_004590.aa.fasta.trans.aa_ali.fasta-gb:  382  (44% of the original 854 positions)
```
